# Capturing water vapors from atmospheric air using superporous gels

**DOI:** 10.1038/s41598-022-08191-3

**Published:** 2022-04-04

**Authors:** Hemant Mittal, Ali Al Alili, Saeed M. Alhassan

**Affiliations:** 1 Department of Mechanical Engineering, Khalifa University of Science and Technology, PO Box 12778, Abu Dhabi, United Arab Emirates; 2 Department of Chemical Engineering, Khalifa University of Science and Technology, PO Box 127788, Abu Dhabi, United Arab Emirates

**Keywords:** Environmental sciences, Engineering, Materials science

## Abstract

Dehumidification performance of most polymer desiccant materials is unsatisfactory because of the complex adsorption mechanism on polymer surface and non-porous structure. A viable alternative of solid desiccants, especially existing polymer desiccants, for capturing water vapors from moist air is the super-porous gels (SPGs). The presence of interconnected channels of pores in its structure facilitates the transfer of water molecules to the internal structure of SPGs. Therefore, in this research work, we are proposing *N*-isopropylacrylamide (NIPAM) and acrylamide (AM) based thermoresponsive SPGs as a potential alternative to the existing conventional solid desiccants. To ensure the formation of interconnected capillary channels, the SPGs were synthesized via gas blowing and foaming technique. Surface morphology of the SPGs was studied using scanning electron microscopy (SEM) and the other physio-chemical characteristics were studied using different techniques like fourier Transform Infrared Spectroscopy (FTIR), X-ray diffraction (XRD) and thermo-gravimetric analysis (TGA). Water vapors adsorption properties of the SPGs were explored via adsorption isotherm and kinetics. The adsorption isotherm was found to be of type-III isotherm with a maximum adsorption capacity of 0.75 g_w_/g_ads_ at 25 °C and 90% relative humidity. Experimental isotherm data correlated well with BET, FHH and GAB isotherm models. Adsorption kinetics suggested that the water vapors diffusion followed intraparticle diffusion and liquid field driving mechanisms collectively. SPGs exhibited very good regeneration and reusability for ten continuous adsorption/desorption cycles. Therefore, the dehumidification efficiency of synthesized SPGs shows that they have potential to replace most of the conventional solid desiccant materials in use.

## Introduction

Different hygroscopic compounds used to capture water vapor from humidity are known as the desiccant materials which can be broadly categorized into solid and liquid desiccants^1^. Different hygroscopic salts like lithium chloride (LiCl) and calcium chloride (CaCl_2_) comes under the category of liquid desiccants^2–4^. These hygroscopic salts have very high water vapors adsorption capacities, but they have a deliquescence property in which they dissolve in the adsorbed water and form crystalline hydrates^5,6^. Therefore, these materials are very difficult to handle and have limited applications. The other class of desiccants is the solid desiccant materials which largely include molecular sieves, silica gel, activated carbon, metal organic frameworks (MoFs), clays, zeolites and polymer desiccant materials^7–10^. The adsorption capacity of solid desiccants is comparatively less as compared to the liquid desiccants, but they are quite stable and easy to handle^1^. Despite the low adsorption capacities, solid desiccants have been used frequently in different industrial and house-hold applications. Solid desiccants play an important role in the packaging industry and air conditioning^11^. Solid desiccant materials capture water vapors from moist air and prevent the damage of products in different industries such as pharmaceutical, electronics, shoes and textile^12^. In packaging industry, most commonly used solid desiccant materials are molecular sieves, clays and silica gel, but they suffer from poor dehumidification efficiencies at certain level of humidity and high desorption temperature which requires large amount of energy to regenerate the desiccants^13,14^.

As an alternative to the conventional solid desiccants, different polymeric materials were investigated^12,15^. However, they have complex adsorption mechanism and in most of the cases, their dehumidification performance is unsatisfactory^13^. The composites of different polymers with hygroscopic salts were also synthesized to enhance their adsorption capacities^1,5,16^. In these composites, the main emphasis was given to avoid the dissolution of deliquescent salts by using the polymer as a matrix to keep the deliquescent salt intact. In most of the cases, the polymeric materials used did not contribute much to the adsorption process and they were used only as a matrix to avoid the dissolution of deliquescent salts^16^. The main problem of these polymer composites is their high regeneration temperature which require a large amount of energy. Therefore, in order to improve the performance of these solid desiccants, novel polymer materials with improved adsorption capacities and low regeneration temperature should be developed.

Recently, super-absorbent gels (SAHs) and their composites with various deliquescent salts have shown very promising results in capturing water from humid air^5,16–18^. SAHs are the crosslinked three dimensional polymers which adsorb water very fast and have swelling capacity upto 100 g_w_/g_ads_^19,20^. SAHs have applications in different fields like water purification^21^ and agriculture^19^. In a study by Xu et al*.*^5^, polymer composites of SAHs with CaCl_2_ salt were used to adsorb water vapors from moist air. In another study, the SAHs of *N*-isopropylacrylamide (NIPAM) were successfully applied to capture atmospheric water^17^. The polymer composites of SAHs with CaCl_2_ salt and carbon nanotubes (CNTs) were also applied for the collection of atmospheric water^16^. It was observed that the adsorption capacity of SAH matrix was only 32% which after incorporation of CaCl_2_ salt and CNTs increased upto 203%. In another study, it was observed that the adsorption capacity of SAHs can be doubled by preparing its composite with 10.6 wt% LiCl solution^1^. In almost all the cases of using polymer composites of SAHs with different salts, the polymer matrix was mainly used to avoid the dissolution of deliquescent salts and the adsorption capacity of bare polymer matrix was low. The low water vapors adsorption capacity of SAHs is mainly due to the highly crosslinked compact structure which restricts the transport of water molecules within the polymer structure. Thus, for fast adsorption kinetics and high adsorption capacity, the polymers should have a porous structure with interconnected capillary channels which will enable the transport of water molecules to the internal structure^22,23^. Therefore, in this work, we are proposing the use of *N*-isopropylacrylamide (NIPAM) and acrylamide (AM) based thermoresponsive super-porous gels (SPGs), having highly porous structure with interconnected capillary channels as a potential alternative solid desiccant to capture water vapors from moist air. SPGs is a very special class of gels having very fast adsorption kinetics and high adsorption capacity^24–26^. Recently, we developed polymer composites of SPGs with AQSOA-Z02 zeolite^27^ and laponite clay^28^ as solid desiccant materials to capture water vapors from moist air. In the present work, SPGs of NIPAM and AM were synthesized using gas foaming and blowing technique and their ability to capture water vapors from moist air was examined at different temperatures. The synthesized SPGs exhibited a dehumidification capacity of 0.75 g_w_/g_ads_ at 90% relative humidity and 25 °C without the use of any deliquescent salt. The high dehumidification efficiency of the synthesized SPGs was studied with regards to adsorption isotherm and adsorption kinetics.

## Experimental

### Materials

NIPAM, AM, ammonium persulfate (APS), 6 N hydrochloric acid (HCl), pluronic F-127 (PF127), tetramethylethylenediamine (TEMED), methylene-bis-acrylamide (MBA) and sodium bicarbonate (NaHCO_3_) were purchased from Sigma Aldrich, UAE. NIPAM was recrystallized using n-hexane whereas all other chemicals were used as received.

### Synthesis of SPGs

For the synthesis of SPGs using NIPAM and AM, the gas blowing and foaming technique was used. Initially, in a three necked round bottom flask a varied amount of monomers, i.e. NIPAM (4.221 to 5.427 mmol) and AM (0.603 to 1.809 mmol), were dissolved in 0.5 mL deionized water with continues stirring and nitrogen bubbling. Thereafter, 1 wt% MBA (200 µL), 10 wt% PF127 (100 µL) and 6 N HCl (60 µL) were added to the reaction flask. 6 N HCl was used as the foaming aid whereas, the foam stabilizer, i.e. PF127, was used to stabilize the foam. When foam stabilizer completely dissolved in the reaction mixture, initiator, i.e. 20 wt% APS (0.1 mL), and accelerator, i.e. 20 v/v% TEMED (0.1 mL), were added. Finally, 0.5 g NaHCO_3_ was added and the mixture was vigorously stirred with a spatula to generate uniform and evenly distributed gas bubbles. The reaction mixture was immediately poured into a square shaped glass mold, covered with parafilm from the top and kept undisturbed for 3 h. Finally, all the samples were freeze dried for 72 h. The molar ratio of NIPAM to AM was changed in the proportion of 9:1, 8:2, 7:3 and 6:4 and labelled as NAM-1, NAM-2, NAM-3 and NAM-4, respectively. After the completion of synthesis and drying, the ability of all the synthesized SPGs to capture water vapors from moist air was initially investigate at two different relative humidity levels, i.e. 70 and 90% at 25 °C, as discussed in “Optimization of NIPAM and AM molar ratio” section. NAM-3 exhibited maximum adsorption capacity amongst all the synthesized SPGs. Therefore, it was used in further studies such as materials characterization and adsorption isotherm and kinetics.

### Materials characterization

The Fourier-transform infrared spectroscopy (FTIR) of NAM-3 was recorded on ATR-FTIR (Bruker, Vertex 70 FTIR spectrophotometer) in the region of 4000–600 cm^−1^ with 4 cm^−1^ resolution for 32 scans. The surface morphology and the presence of interconnected capillary channels in the structure of NAM-3 were studied on Quanta FEG 250 scanning electron microscope (SEM), FEI (USA). All freeze-dried samples were glued to aluminum holder and coated with carbon before SEM analysis. X-ray diffraction patterns (XRD) of NAM-3 before and after washing with deionized water were recorded on X’PERT Powder, PANalytical powder diffractometer (Netherlands) at voltage and current of 40 kV and 40 mA, respectively.

### Water vapors capture experiments

The ability of the synthesized SPGs to capture water vapors from the moist air was investigated using ACS Discovery Environmental chamber. Initially, the adsorption capacities of all the synthesized SPGs were tested at 70 and 90% relative humidity and 25 °C. Before starting these experiments, all the samples were dried at 60 °C in hot air oven. Thereafter, weights of dry samples (*W*_*d*_) were recorded and they were exposed to humidity for 24 h and the weights (*W*_*s*_) were recorded again. The adsorption capacities were calculated using the following expression^28^:

1 $$Adsorption \,capacity= \frac{{W}_{s}- {W}_{d}}{{W}_{d}}.$$

The water vapors adsorption isotherm studies were conducted at 25, 35, 45 and 55 °C having relative humidity between 20 and 90%. For adsorption kinetics, NAM-3 was exposed to 70 and 90% relative humidity at 25 °C. The sample weights were recorded at the dry state and at regular time intervals after exposing them to the humid air. To check the reusability of NAM-3, after complete hydration, the NAM-3 was regenerated at 60 °C and used in the next adsorption cycle. The NAM-3 was hydrated and regenerated for six cycles of adsorption and regeneration.

## Results and discussion

### Optimization of NIPAM and AM molar ratio

**Figure 1:**
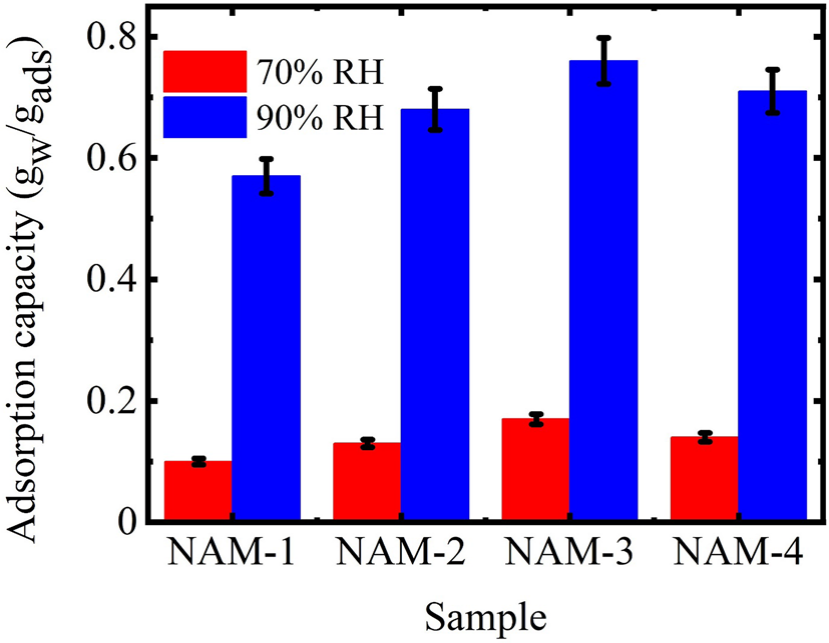
Effect of the molar ratio of NIPAM to AM on the water vapors adsorption capacity at 70 and 90% relative humidity.

Initially, the ability of all the synthesized SPGs with different molar ratio of NIPAM to AM, i.e. in NAM-1 to NAM-4, to capture water vapors were evaluated at 70 and 90% relative humidity at 25 °C (Fig. 1). Primarily, with increasing the concentration of AM in the polymer matrix, i.e. in NAM-1 (with 9:1 molar ratio of NIPAM:AM) to NAM-3 (with 7:3 molar ratio of NIPAM:AM) the adsorption capacity increased from 0.1 to 0.17 g_w_/g_ads_ at 70% relative humidity and from 0.57 to 0.76 g_w_/g_ads_ at 90% relative humidity. This improved adsorption capacity was mainly due to the increased amount of AM in the polymer matrix which supported the adsorption of more water molecules. However, in NAM-4 where the molar ratio of NIPAM to AM was 6:4, adsorption capacity decreased with further increasing AM concentration. In NAM-4, the adsorption capacity reduced to 0.14 and 0.71 g_w_/g_ads_ at 70 and 90% relative humidity, respectively (Fig. 1). This decline in the adsorption capacity in NAM-4 was due to the self-crosslinking between different polymer chains using different functional groups present in AM structure^29^.

### Characterization

**Figure 2:**
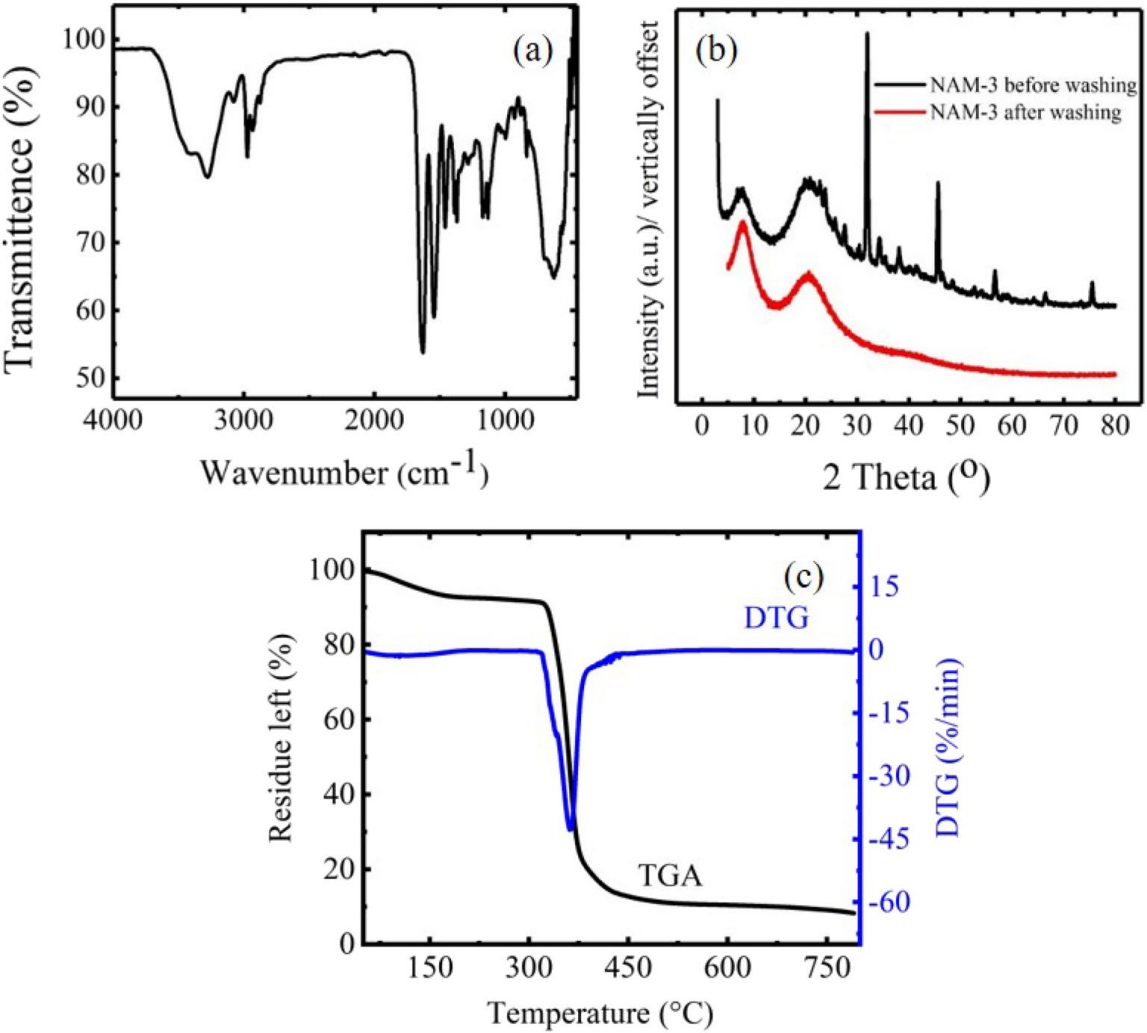
(**a**) FTIR spectrum of NAM-3; (**b**) XRD patterns of NAM-3 before and after washing with water and (**c**) TGA-DTG of NAM-3.

Characteristic peaks of NIPAM and AM were obtained in the FTIR spectrum of NAM-3 (Fig. 2a) at: 3417 cm^−1^ (N–H stretching of amide), 3276 cm^−1^ (–OH stretching of adsorbed water), 3077 cm^−1^ (–NH stretching of secondary amide), 2971 and 2921 cm^−1^(–CH_2_ aliphatic vibrations), 1628 cm^−1^ (C=O stretching vibrations of amide-I), 1550 cm^−1^ (–COO^−^ asymmetric stretching vibrations), 1453 cm^−1^ (NH-in-plane bending of amide-II), 1391 cm^−1^ (–CH bending of isopropyl group), 1281 cm^−1^ (–CN stretching in amide-III), 1170 cm^−1^ (–CO stretching and –OH bending vibrations) and 836 cm^−1^ (–OCN deformation of amide-IV)^30,31^. Presence of these peaks in the FTIR spectrum of NAM-3 confirmed the presence of NIPAM and AM units in SPGs.

Figure 2b shows the XRD patterns of the NAM-3 before and after washing with water. XRD patterns of NAM-3 before washing showed some sharp peaks which were absent in the XRD patterns of NAM-3 after washing. These sharp peaks in the XRD patterns of NAM-3 before washing were because of the presence of sodium ions in the structure^32^. The amorphous nature of NAM-3 was also suggested by the absence of sharp peaks in the XRD pattern of the sample after washing.

The thermogravimetric analysis (TGA) and derivative thermogravimetric (DTG) patterns of NAM-3 was studied in the temperature range of 50–800 °C and is shown in Fig. 2c. The TGA of NAM-3 exhibited two stage decomposition. The first stage decomposition which is associated with the loss of water and other volatile molecules as well as the starting of depolymerisation reactions occurred in the 50–316 °C range with a mass loss of 8.1%. The second and final decomposition stage occurred in the 316–486.3 °C range with 80.1% mass loss. In this decomposition step, the depolymerisation reactions continued and the crosslinks between different polymer chains broke which resulted in the complete collapse of the lightly crosslinked structure of super-porous gels. Derivative thermogravimetric (DTG) curve of NAM-3 is also shown in Fig. 2c. The DTG peak, i.e. *T*_*m*_ associated with the breakage of crosslinks different polymer chains, was observed at 362 °C.

**Figure 3:**
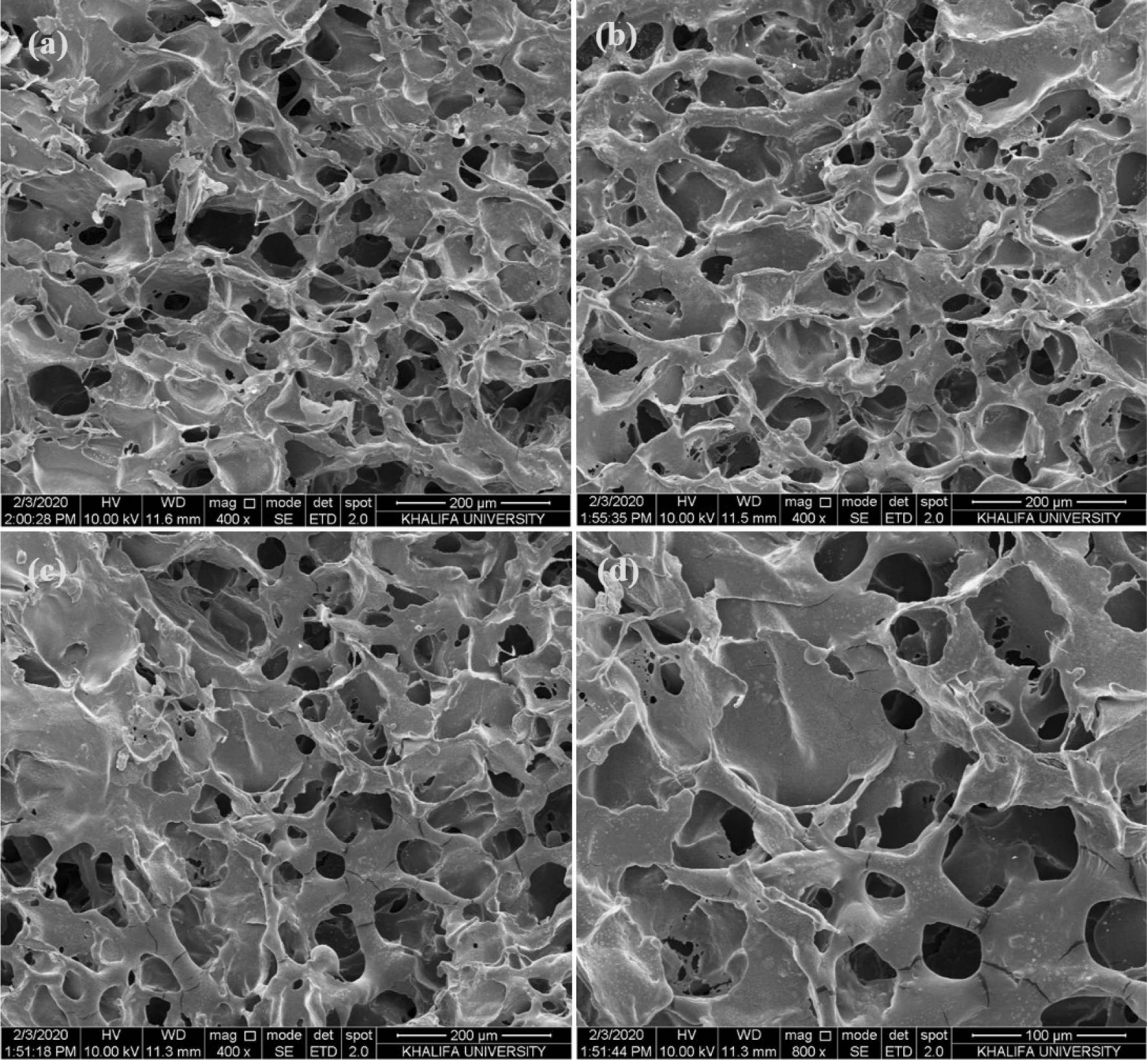
(**a–d**) SEM images of NAM-3 at different magnifications.

It is a well-known fact that the exceptionally high water absorption or swelling properties of SPGs are mainly due to their porous structures. Therefore, the surface morphology and the presence of pores in the structure of NAM-3 was examined using the SEM (Fig. 3a–d). The NAM-3 has porous macrostructure and smooth texture with various interconnected capillary channels of pores generated on the surface. These capillary channels were very well connected which was the main reason for the exceptionally high water uptake capacity and fast kinetics^25,27,28^.

### Adsorption of water vapors from humid air

#### Adsorption isotherm

**Figure 4:**
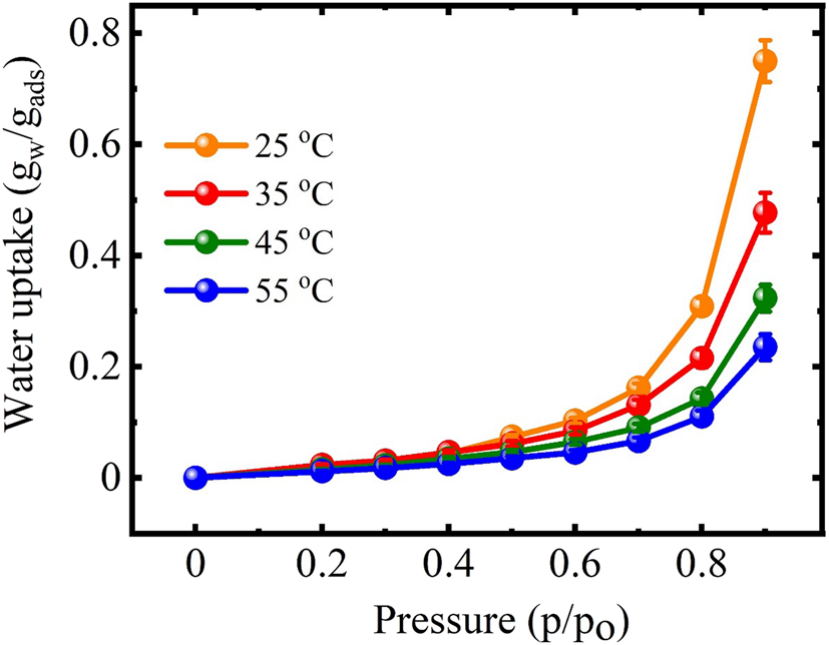
Adsorption isotherm at different temperatures for the NAM-3 in the relative humidity range of 20 to 90%.

Adsorption isotherms for water vapors adsorption using NAM-3 were studied for relative humidity between 20 to 90% at 25, 35, 45 and 55 °C (Fig. 4). The adsorption isotherm at all temperatures was found to follow type-III adsorption isotherm^33,34^, which suggested that this material belongs to the category of desiccants having macroporous structure in which water molecules are generally adsorbed via capillary condensation using the interconnected channels of pores present in its structure^28^. In this type of adsorbents, most of the water molecules after adsorption are present as free water after entrapping in the cavities. However, only a small fraction of the adsorbed water attaches to the surface functional groups of the adsorbent^35^. Existence of type-III adsorption isotherm for NAM-3 was also supported by the SEM images (Fig. 3) which clearly showed the presence of interconnected capillary channels.

The isotherm pattern of NAM-3 can be generally divided into two main regions where in the first region, the adsorption capacity was low and it existed at relative humidity less than 50%, whereas in the second region, the adsorption capacity was very high and it existed when the relative humidity was between 50 and 90%. In the first region (relative humidity < 50%), maximum adsorption capacity was only 0.08 g_w_/g_ads_ at 25 °C and in this region water molecules were mainly attached to the surface functional groups of NAM-3. In this region the adsorption capacity was quite low because the partial pressure of water molecules was not sufficient to penetrate into the polymer structure. Therefore, the water molecules were not able to enter into the internal polymer structure via capillary condensation process^28^. However, in the second adsorption region (relative humidity between 50 and 90%), the NAM-3 exhibited a maximum adsorption capacity of 0.75 g_w_/g_ads_ at 25 °C and 90% relative humidity. The higher adsorption capacity in the second region suggested that with increasing relative humidity, the hydrophilic character of NAM-3 increased and most of the water molecules were adsorbed via capillary condensation process and existed as free water molecules. In this region, with increasing relative humidity, the partial pressure of water molecules increased so they can penetrate deep into the internal structure via capillary condensation process, so in this region the adsorption capacity was very high^36,37^. For all studied temperatures, the slope of the adsorption isotherm was not steep in the lower relative humidity regions which suggested that at the lower humidity values, NAM-3 did not have high affinity for water vapors and it had lower adsorption capacities as compared to other conventional adsorbents such as zeolites and silica gel which generally exhibit type-I adsorption isotherm^38,39^. However, with increasing humidity, the water vapors adsorption of NAM-3 increased and it became much higher than most conventional solid desiccant materials. Further, the adsorption capacity of NAM-3 gradually decreased at elevated temperatures which also suggested that the adsorption of water vapors on NAM-3 was an endothermic process.

**Figure 5:**
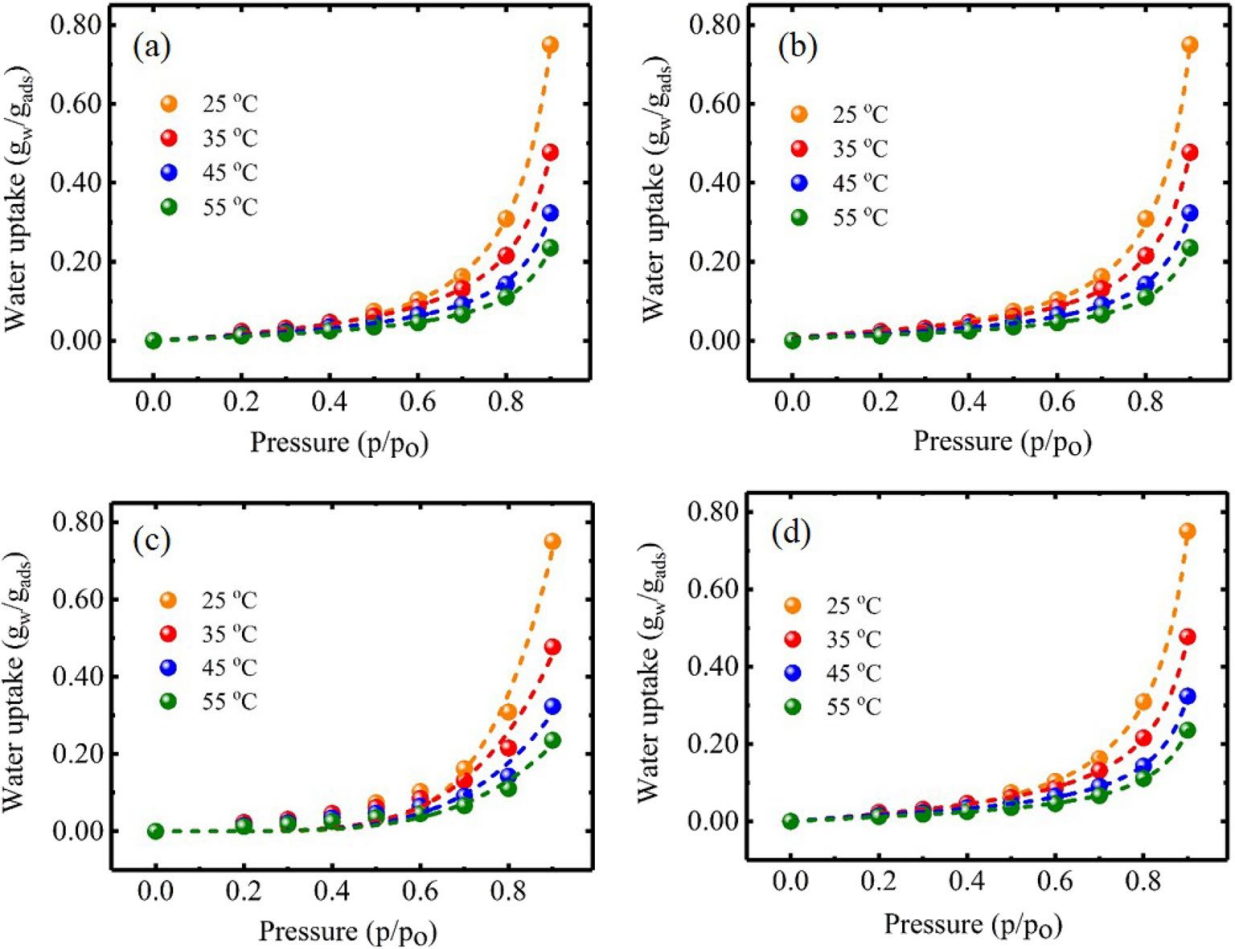
Plot of the fitting of experimental isotherm data to (**a**) BET; (**b**) FHH; (**c**) Freundlich and (**d**) GAB isotherm models.

The adsorption isotherm data was fitted using the non-linear form of different isotherm models such as Brunauer–Emmett–Teller (BET) (Fig. 5a)^40^, Frenkel–Halsey–Hill (FHH) (Fig. 5b)^41^, Freundlich (Fig. 5c)^42^ and Guggenheim, and Anderson and Boer (GAB) models (Fig. 5d)^43^. The theories of these models are provided in the Supplementary Information document and different isotherm parameters calculated are compiled under Table 1. Depending upon the values of different parameters such as AIC (Akaike Information Criterion), BIC (Bayesian Information Criterion) and correlation coefficient (R^2^), the experimental data correlated with the BET, GAB and FHH isotherm models.

**Table 1:** Different isotherm parameters for the water absorption on NAM-3.

Isotherm model	Parameter	Temperature (°C)			
		25	35	45	55
Freundlich	k_F_ (L/mg)^1/n^ (mg/g)	1.39	0.76	0.499	0.360
	n	0.16	0.205	0.216	0.221
	AIC	− 50.67	− 54.13	− 58.81	− 65.59
	BIC	− 54.88	− 58.34	− 63.02	− 69.80
	R^2^	0.975	0.957	0.944	0.950
BET	q_m_ (g_w_/g_ads_)	0.086	0.050	0.033	0.024
	c	0.564	1.51	0.95	2.14
	AIC	− 86.62	− 93.78	− 93.79	− 110.0
	BIC	− 90.83	− 97.94	− 97.99	− 114.27
	R^2^	0.999	0.999	0.999	0.999
GAB	q_m_ (g_w_/g_ads_)	0.076	0.046	0.029	0.023
	c_G_	3.13	2.59	2.03	0.758
	k_G_	1.03	1.00	0.97	0.94
	AIC	− 83.14	− 94.69	− 101.10	− 107.54
	BIC	− 92.35	− 103.91	− 110.31	− 116.75
	R^2^	0.999	0.999	0.999	0.999
FHH	q_m_ (g_w_/g_ads_)	0.045	0.042	0.030	0.023
	r	0.967	0.950	0.925	0.910
	AIC	− 79.33	− 100.05	− 98.36	− 109.55
	BIC	− 83.54	− 104.25	− 102.57	− 113.76
	R^2^	0.999	0.999	0.999	0.999

The adsorption isotherm data fitted quite well with the BET isotherm model (Fig. 5a). This isotherm model generally interprets the multi-layer adsorption isotherms which follow type II and type III isotherms and it is mainly applicable when the water activity, i.e. the value of *p/p*_*o*_, is below 0.45. Therefore, the applicability of BET isotherm predicted that the adsorption was multilayer sorption process and also supported the type-III adsorption isotherm in this particular case^44^. Further, the value of monolayer moisture content (*q*_*m*_) obtained using BET model which represents the amount of water molecules attached to the ionic and polar surface functional groups of the adsorbent via different binding forces also decreased with increasing temperature which predicted that even in the lower humidity regions (particularly below 0.5), the amount of water vapors adsorbed on the surface of NAM-3 decreased at elevated temperatures.

The water vapors adsorption isotherm data of NAM-3 also correlated well with the FHH model (Fig. 5b). This model describes the multilayer adsorption process and assumes that the adsorbate molecules are binded to the adsorbent via weak physical forces of attraction^45^. This isotherm model is mainly used to explain the adsorption of water molecules on different clays. Therefore, it is applicable in this particular case because the mechanism of water vapors adsorption on NAM-3 is very similar to clays. The FHH model is formulated by assuming the presence of adsorption potential gradient which relies on the distance between the surface of adsorbent and adsorbed water layer^46^. Different isotherm parameters obtained from FHH models such as *q*_*m*_ and *r* were found to have lower values at higher temperatures (Table 1) which also supported the lower adsorption capacities at higher temperatures^27^.

The adsorption isotherm data also correlated well with GAB model (Fig. 5d). In case of the adsorbents having interconnected channels of pores, in the lower humidity regions (generally in the region where the relative humidity is lower than 50%), most of the water molecules adsorbed on the outer surface are known as “bounded water” and attached to the surface functional groups mainly by electrostatic interactions^37^. The monolayer adsorption capacity (*q*_*m*_) obtained from GAB model determines the coverage of adsorbent surface by these bounded water molecules. Therefore, for the overall higher adsorption capacity, the value of *q*_*m*_ should be high and for overall lower adsorption capacity, the value of *q*_*m*_ should be low^27^. For the adsorption of water vapors onto NAM-3, the value of monolayer adsorption capacity was higher at lower temperatures which progressively decreased with increasing temperature. That supported the lower adsorption capacity of NAM-3 at elevated temperatures which was further supported by the decrease in the values of *c*_*G*_ and *k*_*G*_ at elevated temperatures (Table 1)^38^.

To check the superiority of NAM-3 for different water vapors adsorption applications, its maximum water vapors adsorption was compared with other reported solid desiccants^16,47–54^ (Table 2). It was observed that NAM-3 has much better adsorption capacity as compared to most solid desiccants materials in use. Further, the regeneration temperature of NAM-3 was low as compared to other adsorbents which will significantly reduce the amount of energy used during the drying or regeneration of the adsorbent and subsequently reduce the cost of the overall process. Most of the conventional solid desiccants used in the water vapors capture applications are expansive as compared to the gels. Further, the synthesis process of gels is can be considered simpler than other solid desiccants such as zeolites, MOFs and clays. Therefore, these results show that the thermos-responsive SPGs of NIPAM and AM possess attractive characteristics that make them good candidates with high potential to replace most of the existing solid desiccants materials in the application of capturing water vapor from moist air.

**Table 2:** Comparison of the water vapors adsorption capacity of NAM-3 with other adsorbents.

Adsorbent	Relative humidity (%)	Temperature (°C)	Adsorption capacity (g_w_/g_ads_)	References
MCM-41	60	24	0.36	Llewellyn et al.^47^
Type-A silica gel	100	25	0.40	Chua et al.^48^
Type-RD silica gel	93	25	0.45	Chua et al.^48^
MCM-48	59	25	0.08	Oh et al.^49^
SBA-1	79	25	0.42	Oh et al.^49^
SBA-15	67	25	0.14	Oh et al.^49^
KIT-1	60	25	0.45	Oh et al.^49^
Zeolite-Y	63	25	0.36	Wisniewski et al.^50^
Zeolite-X	60	23	0.29	Dzhigit et al.^51^
Zeolite-4A	63	25	0.20	Gorbach et al.^52^
Alumina	67	20	0.14	Kim et al.^53^
AA-300 Activated Alumina	60	26	0.18	Desal et al.^54^
PAM hydrogels	80	25	0.32	Li et al.^16^
PAM-CNT	80	25	0.38	Li et al.^16^
NAM-3	90	25	0.75	This study

#### Isosteric heat of adsorption

**Figure 6:**
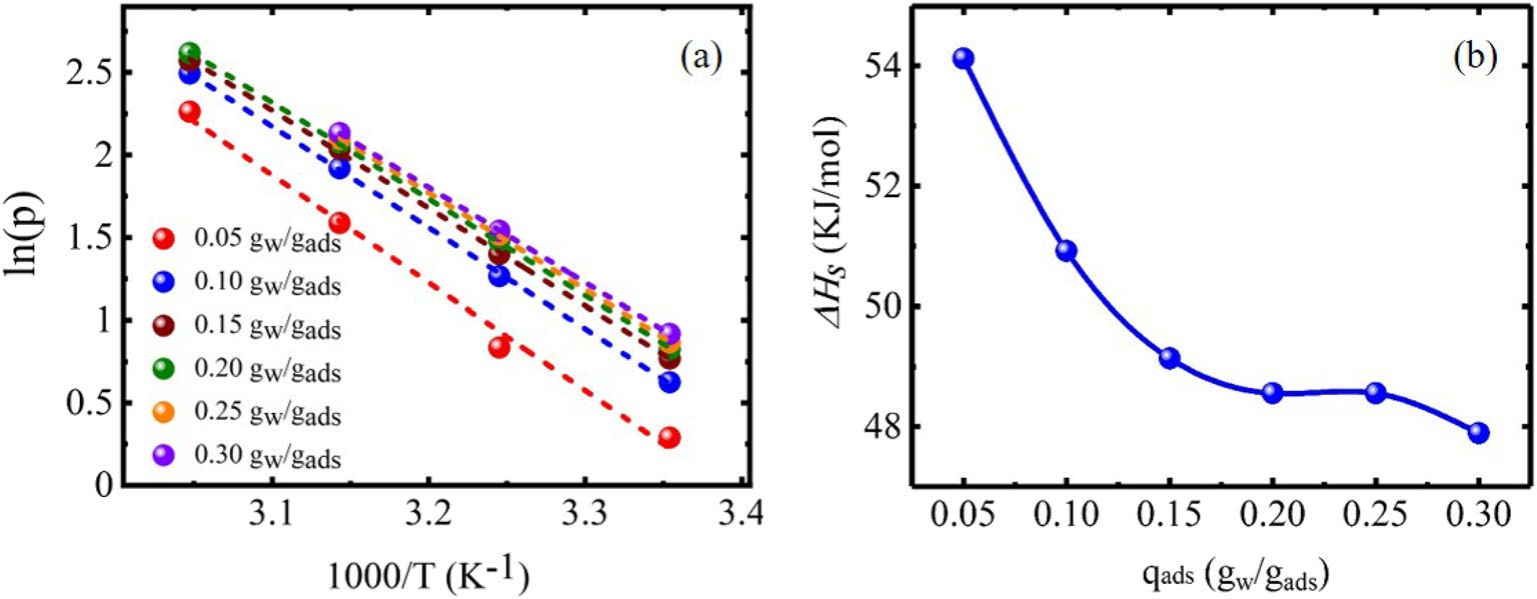
Plots of the (**a**) *ln*(*p*) versus *1/T* and (**b**) the isosteric heat of water vapors adsorption on NAM-3.

The Clausius–Clapeyron equation was used to evaluate isosteric heat of adsorption, i.e. Δ*H*_*s*_. Clausius–Clapeyron equation can be re-presented by the following mathematical equation ^39^:

2 $$ln\left(p\right)=- \frac{{\Delta H}_{s}}{RT}+C,$$

where, *p* (kPa) denotes the water vapor feed pressure, *R* and *T* symbolizes the gas constant and temperature, respectively and *C* denotes the integration constant. For the determination of Δ*H*_*s*_, initially isotherms were converted into isosteres by plotting the isotherms in the form of *ln(p)* vs *1/T* (Fig. 6a) for certain values of the water vapors adsorbed. Then, the isosteric heat of adsorption (Δ*H*_*s*_) was calculated from the slope (− Δ*H*_*s*_/R). The plots of Clausius–Clapeyron equation representing Δ*H*_*s*_ at different adsorption capacities is shown in Fig. 6b and values of Δ*H*_*s*_ along with correlation coefficients are tabulated in Table 3.

**Table 3:** Estimation of isosteric heat and other fitting parameters for the water vapors adsorption on NAM-3.

q_ads_ (g_w_/g_ads_)	− Δ*H*_*s*_/R (K)	Δ*H*_*s*_ (kJ/mol)	R^2^
0.05	− 6.51	54.12	0.989
0.1	− 6.124	50.91	0.999
0.15	− 5.91	49.13	0.999
0.2	− 5.84	48.55	0.999
0.25	− 5.84	48.55	0.999
0.3	− 5.76	47.88	0.999

The values of Δ*H*_*s*_ were comparatively higher at the lower adsorption capacities because at lower humidity values, the water molecules were attached to the surface functional groups of NAM-3 through strong binding forces and mostly presented as bounded water, so the Δ*H*_*s*_ values were higher. However, with increasing relative humidity, the partial pressure of water molecules increased and they started penetrating deeper into the internal structure of adsorbent, so most of the water molecules were present as free molecules and the values of Δ*H*_*s*_ were comparatively low^28^. The Δ*H*_*s*_ values of NAM-3 were in the range of 47–54 kg/mol (Table 3). Further, at higher adsorption capacities, the Δ*H*_*s*_ value of NAM-3 was comparable with water (44 kJ/mol) which suggested that the water clusters started forming and filling the pores of adsorbent with water molecules once the monolayer formation completed^39^.

#### Kinetics of adsorption

**Figure 7:**
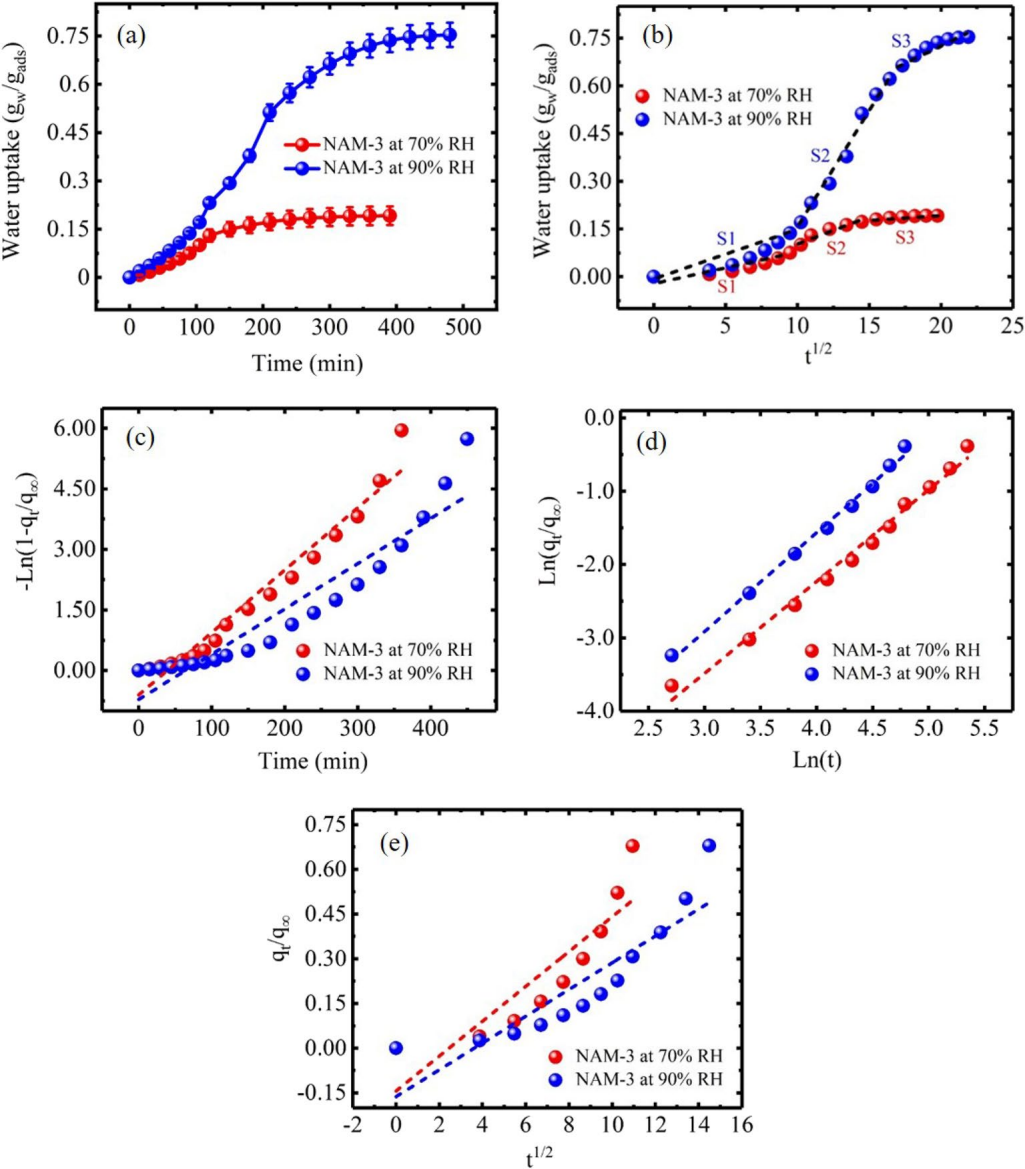
Plots of (**a**) water vapors adsorption kinetics; (**b**) intraparticle diffusion model; (**c**) linear driving force kinetics model; (**d**) *ln*(*q*_*t*_*/q*_*∞*_) versus *ln*(*t*) and (**e**) *q*_*t*_*/q*_*∞*_ versus *t*^1/2^ for the adsorption of water vapors on NAM-3 at 25 °C and 70 and 90% relative humidity.

Kinetics of the water vapors adsorption onto NAM-3 was studied at two different relative humidity values of 70 and 90% (Fig. 7a). At both relative humidities, the rate of water vapors adsorption was slow at the beginning, then increased for some time, and finally became slow while proceeding towards equilibrium. The slower adsorption rate in the beginning might be because of the fact that the surface of NAM-3 was hard and most of the pores of the gel polymer were close. However, after adsorbing water molecules, the pores of gel structure started opening and adsorbing water molecules via capillary condensation process, so the rate of water vapors adsorption increased. Further, the adsorption of water vapors became slower while approaching equilibrium because most of the cavities in the gel polymer structure were occupied by the water molecules and it was difficult for new molecules to enter the polymer structure.

The complete process of the water vapors adsorption on a gel polymer is a combination of different steps: firstly, the water molecules were adsorbed onto the outer boundary layer of gel followed by the subsequent transfer of water molecules to the inner boundary layer and finally the diffusion into the internal structure of gel via capillary condensation process. The slowest step in the whole process determines or controls the overall rate of the adsorption process. Therefore, to determine the rate controlling step, experimental kinetics data was fitted to the intraparticle diffusion (Fig. 7b) and linear driving force (LDF) (Fig. 7c) models. Intraparticle diffusion model can be represented as^55^:

3 $${q}_{t}= {k}_{ID}{t}^\frac{1}{2} +C,$$

where, *q*_*t*_ is adsorption capacity at time *t*. If rate of adsorption was only controlled by the intraparticle diffusion mechanism, then the plot of this model should not have a slope and the plot must pass through the origin^24^. However, the plot of intraparticle model was found to have three different and individual sections, i.e. S1, S2 and S3, with distinct slopes. Therefore, intraparticle diffusion did not controlled the diffusion mechanism alone, but there might be contribution of other mechanisms. Different kinetics parameters calculated using intraparticle diffusion model for regions S1, S2 and S3 are compiled in Table 4.

**Table 4:** Different kinetics parameters calculated using Intraparticle diffusion model.

	k_ID_ (mg/g min^−1/2^)	C (mg/g)	R^2^
**70% Relative humidity**			
S1	0.0077	0.013	0.837
S2	0.0095	0.097	0.991
S3	0.0014	0.179	0.991
**90% Relative humidity**			
S1	0.044	0.083	0.901
S2	0.050	0.095	0.874
S3	0.012	0.062	0.979

Experimental data was further fitted to the LDF model, Fig. 7c. The LDF can be represented by the following mathematical equation^56^:

4 $${q}_{t}= {q}_{\infty }\left(1-{e}^{-{k}_{eff}t}\right).$$

Taking natural log and rearranging:

5 $$ \ln \left( {1 - q_{t} /q_{\infty } } \right) = k_{{eff}} t,$$

where, *q*_*∞*_ represents equilibrium adsorption capacity, *k*_*eff*_ (1/min) represents mass-transfer coefficient. The slope of the LDF model plot, i.e. − *ln* (1* − q*_*t*_*/q*_*∞*_) vs *t*, provides *k*_*eff*_ values. In this case, the plot of LDF model had slope which ruled out the possibility of the diffusion mechanism solely controlled by LDF. The value of *k*_*eff*_ was higher at 70% as compared to the 90% relative humidity, Table 5. The higher values of *k*_*eff*_ at 70% relative humidity suggested that the adsorption equilibrium was achieved much faster at 70% as compared to 90% relative humidity. At 70% relative humidity, water molecules were mainly attached to the NAM-3 surface, therefore, adsorption equilibrium was attained quicker. On the other hand, at higher relative, i.e. at 90% relative humidity, the water molecules had sufficient pressure to occupy the cavities of the gel polymer via capillary condensation process. Therefore, it took comparatively more time to attain the adsorption equilibrium. Moreover, at higher humidity values, the water molecules are mainly adsorbed via gel bonding interactions and present in the cavities of gel polymer as free water molecules. However, at lower humidity values, the water molecules are attached to the surface functional groups of NAM-3 via stronger adsorbate-adsorbent interactions. Therefore, the *k*_*eff*_ values were higher at 70% as compared to 90% relative humidity^39^.

**Table 5:** Different kinetics parameters of NAM-3 at 70 and 90% relative humidity.

Samples	Relative humidity (%)	Swelling kinetics parameters				
		First order	R^2^	k	n	D (cm^2^/cm)
		*K*_*eff*_ (1/min)				
NAM-3	70	0.015	0.954	7.25E−4	1.251	1.65E−4
NAM-3	90	0.011	0.893	9.59E−4	1.346	9.49E−5

The diffusion mechanism of water molecules was also studied using 60% diffusion method using the following equation^56^:

6 $$\frac{{q}_{t}}{ {q}_{\infty }}= {{k}_{1} t}^{n}.$$

Taking natural log on both sides,

7 $$ln\left(\frac{{q}_{t}}{{q}_{\infty }}\right)=k+n ln\left(t\right).$$

The nature of diffusion mechanism can be determined from the value of *n* which was calculated from the slope of the plot of *ln*(*q*_*t*_*/q*_*∞*_) and *ln*(*t*), Fig. 7d. The value of *n* at both relative humidity values was higher than 1.0 which suggested that NAM-3 followed case-II type diffusion mechanism^56^.

The short time fractional uptake method was used to calculate the intercrystalline diffusion coefficient (*D*) by using the following equation^19^:

8 $$ \frac{{q_{t} }}{{q_{\infty } }} = 4\left( {Dt/\pi r^{2} } \right)^{{1/2}}  - \pi \left( {Dt/\pi r^{2} } \right) - \frac{\pi }{3}\left( {Dt/\pi r^{2} } \right)^{{3/2}} , $$

where, *r* is the polymer radius.

Assuming *n* = 0.5 and comparing Eqs. (6) and (8):

9 $$ k = 4\left( {D/\pi r^{2} } \right)^{{1/2}}. $$

10 $$ \frac{{q_{t} }}{{q_{\infty } }} = 4\left( {Dt/\pi r^{2} } \right)^{{1/2}}. $$

Values of *D* were obtained from the plot of *q*_*t*_*/q*_*∞*_ vs *t*^1/2^ as slope, Fig. 7e, which are compiled in Table 5. The *D* value was higher at 70% than 90% relative humidity which also supported the fast attainment of adsorption equilibrium at 70% relative humidity^28^.

### Regeneration and reusability

**Figure 8:**
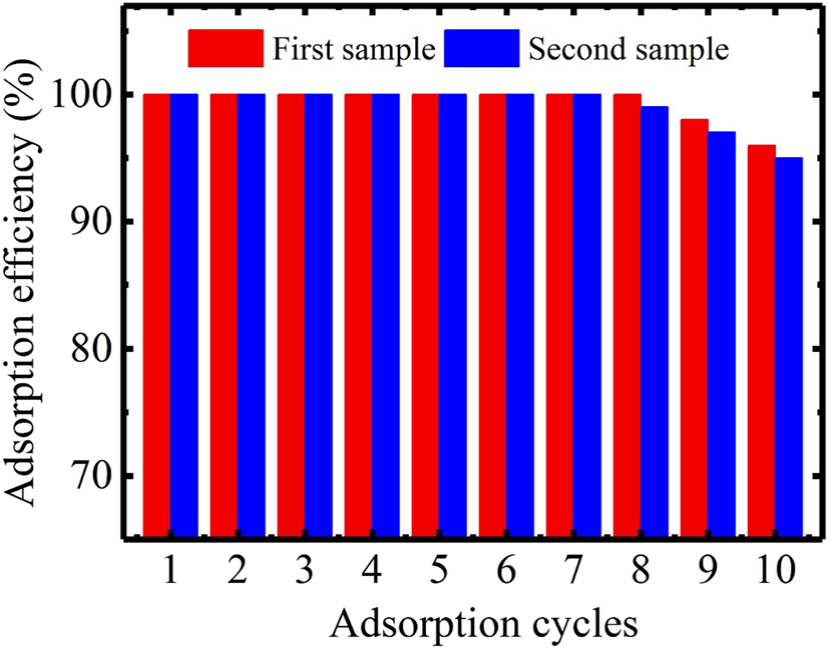
Successive water vapors adsorption for continuous ten cycles using NAM-3.

Besides the good adsorption capacity, the overall performance of any adsorbent material also depends upon its recyclability and the ability to be used for multiple cycles of adsorption/desorption. Therefore, the reusability of NAM-3 was tested for ten continuous adsorption/desorption cycles. Initially, the water vapors were adsorbed on NAM-3 at 90% relative humidity and after attaining the equilibrium adsorption state, the fully hydrated NAM-3 was regenerated at 60 °C and used again for water vapors adsorption. The recyclability experiments of NAM-3 were duplicated by using two similar samples of NAM-3, Fig. 8. In the first sample, no decline or reduction in the adsorption capacity was observed for the first eight cycles of adsorption. However, in the second sample, the adsorption capacity was remained for the first seven cycles. In the tenth cycle, both samples exhibited very good reuse efficiency with 96% for the first sample and 95% for the second one. The small reduction in the adsorption efficiency after continuous use of NAM-3 was due to the mechanical breakdown of polymer structure^28^.

## Conclusions

NIPAM and AM based highly porous SPGs were synthesized successfully. The SEM images confirmed that it had interconnected capillary channels of pores which were responsible for the high adsorption capacity and fast kinetics. The synthesized SPGs were used as solid polymer desiccant materials to capture water vapors from moist air. NAM-3 was found to have the highest adsorption capacity among all the synthesized SPGs. NAM-3 exhibited a high adsorption capacity of 0.75 g_w_/g_ads_ at 25 °C and 90% relative humidity. The adsorption isotherm followed type-III isotherm and the experimental data correlated well with BET, GAB and FHH isotherm models. The results suggested that the high adsorption capacity was mainly because of the existence of interconnected capillary channels of pores in its structure. Adsorption capacity of NAM-3 exhibited lower adsorption capacity at higher temperatures suggesting that the adsorption of water vapors onto NAM-3 was an endothermic adsorption process. Further, the isosteric heat of adsorption decreased with increasing adsorption capacities suggesting that most of the water molecules were adsorbed via capillary condensation process and existed in the cavities of NAM-3 as free water molecules at high humidity values. The adsorption kinetics predicted that the diffusion of water molecules into the polymer structure followed type-II diffusion mechanism. Furthermore, NAM-3 showed excellent cyclability and it was continuously used for ten cycles of adsorption/desorption. Therefore, it can be concluded that the synthesized solid desiccant material of NIPAM had exceptionally high water vapors adsorption capacity and it can adsorb high amount of water without using other hygroscopic materials such as deliquescent salts, zeolites, clays and silica gel. The successful utilization of these SPGs to capture the water vapors from moist air opened the doors of a new exciting class of solid desiccants.

## Supplementary Information

Supplementary Information.
