# Supplementary material for: Capturing water vapors from atmospheric air using superporous gels
Source: Sci Rep. 2022 Apr 4;12:5626. doi: 10.1038/s41598-022-08191-3 (PMC8980045; doi:10.1038/s41598-022-08191-3)
Supplement: Supplementary file 1 — Supplementary Information. [file 41598_2022_8191_MOESM1_ESM.docx]

**Supporting Information**

**Capturing water vapors from atmospheric air using superporous gels**

Hemant Mittal^1^, Ali Al Alili^1*^, Saeed M Alhassan^2*^

^1^ Department of Mechanical Engineering, Khalifa University of Science and Technology, PO Box 12778, Abu Dhabi, United Arab Emirates

^2^ Department of Chemical Engineering, Khalifa University of Science and Technology, PO Box 127788, Abu Dhabi, United Arab Emirates

*Corresponding author E-mail: [ali.alalili@ku.ac.ae](mailto:ali.alalili@ku.ac.ae) (Ali Al Alili);

[saeed.alkhazraji@ku.ac.ae](mailto:saeed.alkhazraji@ku.ac.ae) (Saeed M Alhassan)

**Water vapor adsorption isotherm models**

**Freundlich isotherm**

Freundlich adsorption isotherm generally represents a multilayer reversible adsorption. The mathematical expression of Freundlich isotherm is an exponential equation which can be represented as:

|  | $q= {k_{F}\left( \frac{p}{p_{O}} \right)}^{\frac{1}{n}}$ | $(1)$ |
| --- | --- | --- |

where, *q* is the water adsorbed, *k_F_* represents Freundlich isotherm constant, *p/p_o_* is relative humidity or pressure and *n* is adsorption intensity refers to the heterogeneous adsorbent.

**BET (Brunauer, Emmett and Teller) isotherm**

The BET adsorption isotherm is the most widely studied and applied theoretical adsorption isotherm model, but it is only applicable when the value of *p/p_o_* lies between 0.1 and 0.3. The mathematical equation of BET model is represented as:

|  | $q= \frac{q_{m}ca_{w}}{\left( 1-a_{w} \right)\left( 1+\left( c-1 \right)a_{w} \right)}$ | $(2)$ |
| --- | --- | --- |

where, a_w_ = *p/p_o_* and represents water activity; *q_m_* represents maximum monolayer adsorption capacity and *c* represents BET constant related to the heat of adsorption.

**GAB (Guggenheim, Anderson and Boer) isotherm**

The GAB model developed by Guggenheim, Anderson and De-Boer is based on the theory of multilayer gas adsorption and it is basically an extension of BET model which can be applied over a much wider range of pressure, i.e. 0.1 ≤ *p/p_o_* ≤ 0.9. The mathematical equation of GAB model can be written as:

|  | $q= \frac{q_{m}c_{G}k_{G}a_{w}}{\left( 1-k_{G}a_{w} \right)(1-k_{G}a_{w}+ c_{G}k_{G}a_{w} )}$ | $(3)$ |
| --- | --- | --- |

where, *c_G_* and *k_G_* represent GAB constants related to multilayer and monolayer adsorption. BET and GAB isotherms become identical when the value of GAB constant *k_G_* becomes unity.

**FHH (Frenkel-Halsey-Hill) isotherm**

FHH model developed by Frenkel, Halsey and Hill generally represent multilayer adsorption on the flat solid surface and capillary condensation. The mathematical equation of FHH model can be represented as:

|  | $q= {q_{m}\left[ -ln\left( \frac{p}{p_{O}} \right) \right]}^{\frac{-1}{r}}$ | $(4)$ |
| --- | --- | --- |

where, *r* is FHH constant and *1/r* represents nature of interactions between the solid surface i.e. adsorbent, and the vapor or gas. Normally, the value of *r* for water vapor adsorption on graphite is almost unity. If the value of *r* is very large, then the interactions between the solid surface and vapor are very specific and limited to the neighborhood of solid surface only. However, if *r* has a small value, then the interactive forces between solid surface and water vapor are mainly van der Waals forces which can extend up to a comparatively larger distance.
